# Dissecting the infodemic: An in-depth analysis of COVID-19 misinformation detection on X (formerly Twitter) utilizing machine learning and deep learning techniques

**DOI:** 10.1016/j.heliyon.2024.e37760

**Published:** 2024-09-12

**Authors:** Asma Ul Hussna, Md Golam Rabiul Alam, Risul Islam, Bader Fahad Alkhamees, Mohammad Mehedi Hassan, Md Zia Uddin

**Affiliations:** a Department of Computer Science and Engineering, BRAC University, Dhaka, Bangladesh; b Palo Alto Networks Inc., Santa Clara, CA, USA; c Department of Information Systems, College of Computer and Information Sciences, King Saud University, Riyadh, Saudi Arabia; d Sintef Digital, Oslo, Norway

**Keywords:** COVID-19, Fake news, Community analysis, Twitter, Disseminator, Misinformation, Social network analysis, Deep learning, Machine learning

## Abstract

The alarming growth of misinformation on social media has become a global concern as it influences public opinions and compromises social, political, and public health development. The proliferation of deceptive information has resulted in widespread confusion, societal disturbances, and significant consequences for matters pertaining to health. Throughout the COVID-19 pandemic, there was a substantial surge in the dissemination of inaccurate or deceptive information via social media platforms, particularly X (formerly known as Twitter), resulting in the phenomenon commonly referred to as an “Infodemic”. This review paper examines a grand selection of 600 articles published in the past five years and focuses on conducting a thorough analysis of 87 studies that investigate the detection of fake news connected to COVID-19 on Twitter. In addition, this research explores the algorithmic techniques and methodologies used to investigate the individuals responsible for disseminating this type of fake news. A summary of common datasets, along with their fundamental qualities, for detecting fake news has been included as well. For the purpose of identifying fake news, the behavioral pattern of the misinformation spreaders, and their community analysis, we have performed an in-depth examination of the most recent literature that the researchers have worked with and recommended. Our key findings can be summarized in a few points: (a) around 80% of fake news detection-related papers have utilized Deep Neural Networks-based techniques for better performance achievement, although the proposed models suffer from overfitting, vanishing gradients, and higher prediction time problems, (b) around 60% of the disseminator related analysis papers focus on identifying dominant spreaders and their communities utilizing graph modeling although there is not much work done in this domain, and finally, (c) we conclude by pointing out a wide range of research gaps, for example, the need of a large and robust training dataset and deeper investigation of the communities, etc., and suggesting potential solution strategies. Moreover, to facilitate the utilization of a large training dataset for detecting fake news, we have created a large database by compiling the training datasets from 17 different research works. The objective of this study is to shed light on exactly how COVID-19-related tweets are beginning to diverge, along with the dissemination of misinformation. Our work uncovers notable discoveries, including the ongoing rapid growth of the disseminator population, the presence of professional spreaders within the disseminator community, and a substantial level of collaboration among the fake news spreaders.

## 1 Introduction

The SARS coronavirus 2 (SARS CoV 2), currently prevalent worldwide, is responsible for causing the coronavirus disease, which we all known as COVID-19 [1]. The virus was initially identified in Wuhan, Hubei, China, and rapidly disseminated worldwide in a remarkably short period of time. On January 30, 2020, the new coronavirus outbreak was proclaimed a public health emergency. Subsequently, on March 11, 2020, it was officially recognized as a pandemic [1]. COVID-19 has a major impact on the average longevity of the individuals who are affected. It induces a severe form of acute respiratory syndrome, mostly through its highly infectious mode of transmission. There is widespread global interest and engagement in gaining knowledge and understanding regarding the facts behind COVID-19. Presently, the prevailing COVID-19 situation has deteriorated due to a substantial reliance on information disseminated through social media platforms. The general public obtains COVID-19 case statistics, guidance, and healthcare information, including emotional and inspiring videos, from various social networking websites. The individuals willingly embrace the information regarding this subject without any hesitation or uncertainty. Therefore, despite the worldwide recovery from the COVID-19 pandemic, worries surrounding the dissemination of false or misleading information related to the virus have not diminished. Due to the seamless efforts of the misinformation spreaders, the COVID-19 pandemic has experienced an increase in the dissemination of false information, which has been further strengthened through the internet, resulting in much more severe repercussions. However, social media has become a formidable medium of communication, which makes it an easy target for misinformation spreaders who use it as a medium of misinformation dissemination.

Twitter is a highly renowned social media network with around 528.3 million active users [2]. Along with other social media platforms, it has faced severe criticism for its involvement in facilitating the dissemination of false information and controversies, particularly in relation to the COVID-19 pandemic. There is an increasing apprehension that the confluence of fake information and disinformation has worsened the global spread of misleading information, despite the substantial impact that social media has had on raising awareness. Regrettably, there are individuals who are disseminating false and lethal information via social media, exploiting the current pandemic circumstances. An instance of misinformation is the claim that the ‘Ivermectin’ tablet can serve as a remedy for COVID-19. A multitude of individuals have unwavering faith in this drug and do not hesitate to consume it. However, the truth is that ‘Ivermectin’ is not an antiviral medication but rather is commonly employed for the treatment of parasitic illnesses [3]. The dissemination of such deceptive information not only jeopardizes the physical well-being of the general population but also poses a significant risk to their mental health [4]. The emergence of the COVID-19 coronavirus and its worldwide ramifications on education, economics, and social interactions, as well as the mental well-being of the general public, youth, and students [5], [6], [7].

While many individuals see social media content as authentic, it is very common for certain individuals to exploit this platform for personal gain by disseminating false information. There is a vast number of tweets pertaining to COVID-19, and not all of them possess accuracy or truthfulness. From the very early stage of the COVID-19 pandemic, researchers have come up with their studies to investigate the detection of COVID-19-related disinformation on Twitter, its method of spreading, and the community responsible for disseminating such fake information. This review paper aims to concentrate on popular research models in the fields of machine learning and deep learning that have the ability to reliably recognize fake news. Subsequently, it delves into employing a social network analysis model to comprehend the intricacies of the individuals responsible for disseminating misinformation. Hence, we examine some widely used datasets pertaining to COVID-19, along with their respective methodologies. In summary, this paper encompasses two broad research thrusts: (a) detection of fake news using traditional machine learning and deep learning models, and (b) examination of the dynamics of misinformation disseminators. Along with the above-mentioned research thrusts, we address two basic research questions, which we elaborate in the next section. Our main contributions to this paper are as follows:

a) We gather, synthesize, and perform a comprehensive analysis of 87 highly related research publications from a misinformation-related corpus of 600 over the period of the last five years.
b) We discuss the methodological approaches and critically evaluate the strengths and weaknesses of selected studies, identifying research gaps in knowledge, and finally,
c) We suggest a wide range of potential future research directions to mitigate the research gaps, for example, utilizing a large training dataset. Going one step further, we have built a large database of training datasets by compiling the datasets from 17 research works. The fellow researchers can easily merge these datasets and use that to train the state-of-the-art models to detect fake news.

We highlight the key findings in the following points:

a) In the domains of community analysis and the detection of fake news, deep learning algorithms and graph mining techniques have proven to be extraordinarily effective and are positioned to bring about a paradigm shift.
b) The ecosystem responsible for propagating fake news connected to COVID-19 is significant and highly collaborative, and its dynamics continue to expand even after the pandemic period.
c) There is a huge research gap in the domain of tracking the activities of misinformation spreaders across online and social media platforms.

The remaining sections are organized as follows: Review methodology is detailed in Section 2. To gain a comprehensive understanding of the essential AI/ML and DL approaches required to construct a fake news detection model, refer to Section 3. Section 4 delves into a comprehensive analysis of the methods used by those who spread fake news. Section 5 outlines the primary discoveries and the possible fake news dataset areas for future investigation in combating COVID-19, while Section 6, 7 provides the concluding observations, discussion, and recommendations for future research.

## 2 Review methodology

Section 2 outlines the systematic review methodology that we employ for our investigation. The research questions are developed to facilitate the discovery of relevant academic literature. Additionally, we offer a comprehensive examination of various information sources and conduct a detailed review of papers yearly. Subsequently, we address the literature search approach, search terms, paper selection process, and criteria for the inclusion and exclusion of relevant papers to determine the ultimate selection of research articles.

### 2.1 Research questions formulation

After performing an initial assessment of the latest literature, the research scope, research questions, and inclusion/exclusion criteria had been determined. Subsequently, we generate and address the research questions for this work. To keep interested readers well-informed, we present the research questions below:

– *RQ1: How can we identify COVID-19-related fake news more accurately?*

– *RQ2: How can we initiate our comprehension of the individuals who disseminate fake information and their patterns of collaboration?*

### 2.2 Sources of information

**Figure 1:**
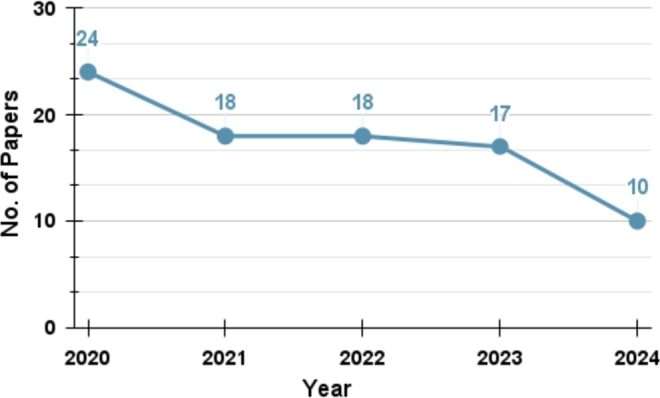
Number of papers vs Year plot shows the number of papers we select from each year.

To gather the essential knowledge within our scope, we carry out an extensive search from scientific publications in journals and conferences, as well as books and other media. This study makes use of various renowned academic resources and digital libraries. The search platforms we encompass include, but are not limited to, Google Scholar, IEEE Xplore, Springer, Elsevier, Scopus, ACM Digital Library, PubMed, Researchgate, Semantic Scholar, etc. We opt to include the most recent related papers in our study, which is exhibited in Fig. 1. The Figure indicates our intention of including the most recent as well as important papers in our study. A significant number of 24 papers have been collected since 2020. This is since most of the COVID-19-related Twitter dataset was generated in 2020, which is the peak pandemic time. Additionally, we show the number of research articles collected from various online search platforms in Table 1. The table indicates that a substantial number of relevant papers have been collected from IEEE.

**Table 1:** Online search platforms and the quantity of research papers selected from each online platform are presented.

Online Resources	No. of Papers
IEEE	14
Springer	8
ACM digital library	7
MDPI	5
Elsevier ScienceDirect	6
JMIR	5
ArXiv	14
Others	28

### 2.3 Search strategy

We have concentrated on including research articles over a period of five consecutive years. However, we use a rich set of highly correlated search keywords to extract the relevant research articles from numerous online publication-oriented platforms. A concentrated version of the search keywords used is detailed in Table 2.

**Table 2:** An index of the search terms used to find the relevant material.

Search Keywords
‘Twitter COVID-19’ and ‘Fake news’ and ‘Machine Learning’ and ‘Prediction’ or ‘Detection’
‘Twitter COVID-19’ and ‘Fake news’ and ‘Deep Learning’ and ‘Prediction’ or ‘Detection’
‘Twitter COVID-19’ and ‘Fake news’ and ‘Artificial Intelligence’ and ‘Prediction’ or ‘Detection’
‘Twitter COVID-19’ and ‘Fake news’ and ‘Neural Network’ and ‘Prediction’ or ‘Detection’
Deep Learning and ‘Twitter’ and ‘CoV2’ or ‘Coronavirus’ or ‘COVID-19’
‘Machine Learning’ and ‘Twitter’ and ‘CoV2’ or ‘Coronavirus’ or ‘COVID-19’
‘Prediction’ and ‘Outbreak prediction’ and ‘ML’ and ‘AI’ and ‘CoV2’ or ‘Coronavirus’ or ‘COVID-19’
‘Fake news’ and ‘Twitter COVID-19’ and ‘Disseminator’ or ‘Community analysis’
‘Disseminator’ or ‘Community pattern’ and ‘Twitter COVID-19’ and ‘Fake news’
‘Twitter COVID-19’ and ‘Fake news’ and ‘Spreaders’ and ‘Network analysis’
‘Disseminator’ or ‘Community’ and ‘Twitter COVID-19’ and ‘Fake news’
‘Twitter COVID-19’ and ‘Fake news’ and ‘Spreaders’ and ‘Prediction’ or ‘Detection’

### 2.4 Study selection, exclusion and inclusion criteria

A methodical search to find the relevant research publications has been conducted using online sources of publication. We discover a conclusive collection of 87 articles to be included in this work. We utilize a set of inclusion and exclusion criteria to select the final research publications from the initial aggregation of research papers. Scrutinizing the abstract, we dismiss the manuscripts that do not satisfy the filtering criteria. We only select the most vital articles that would improve our comprehension of fake news. We utilize these vital 87 papers to acquire knowledge on the subject, elucidate the difficulties, evaluate the detection strategies, and deliberate on probable future directions in the rest of this review paper.

**Figure 2:**
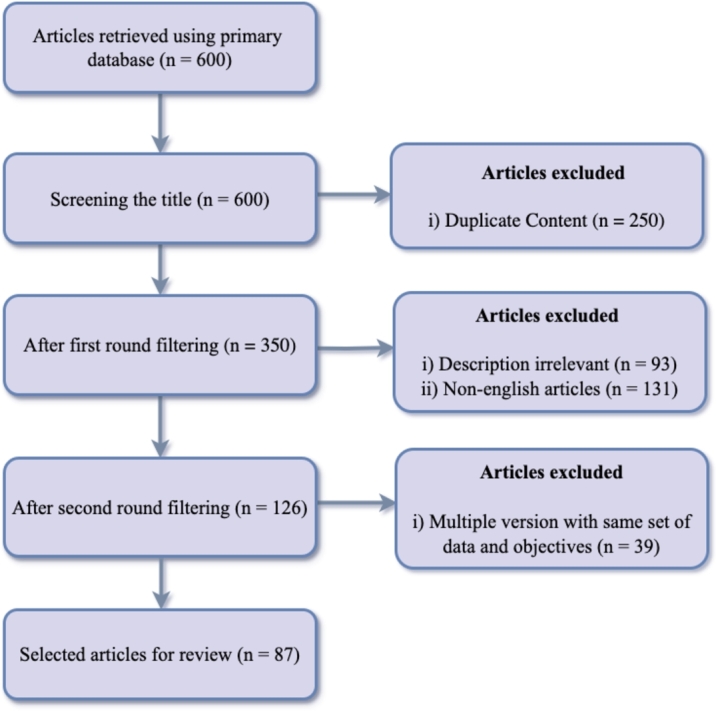
The Prisma flowchart shows the process of inclusion and exclusion of research articles in this study, where n denotes the number of articles.

Fig. 2 presents a traditional Prisma flowchart that illustrates the article selection process. The process encompasses many steps that adhere to the exclusion-inclusion criteria. The initial search utilizing the search keyword yields a total of 600 research papers from the specified online publication portals. Out of these 600 papers, the research selection procedure began by eliminating duplicate articles, resulting in the exclusion of 250 articles during the first screening phase. Subsequently, only the publications in the English language are evaluated by examining their abstracts, and occasionally the introductions, to determine their suitability according to the inclusion criteria. After the completion of the second phase of the selection process, a grand total of 126 articles are deemed eligible. During the third round, we eliminate another 39 articles because newer versions of these studies have already been published using the same data and investigating the same objective. Following the inclusion-exclusion methodology in three distinct phases, a cumulative sum of 87 academic papers is deemed eligible for inclusion in this review work.

## 3 COVID-19 fake news prediction on Twitter

**Figure 3:**
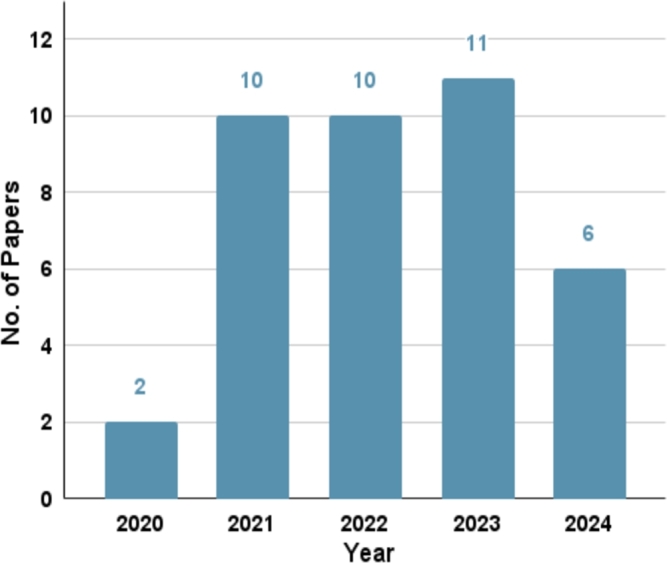
The plot shows the number of fake news detection-related papers selected in this review paper per year.

We have experienced a distressing COVID-19 pandemic phase, characterized by a lethal spike of cases that has rapidly spread worldwide. A multitude of distinguished researchers have diligently worked towards eradicating the COVID-19 infodemic and propose a plethora of approaches for identifying spurious information pertaining to COVID-19. This section focuses on popular artificial intelligence and machine learning models, fusion models, and deep learning models in relation to their accuracy in detecting fake news on Twitter. We have selected a bunch of mostly cited and relevant articles spanning from 2020 to 2024 on this topic. Fig. 3 displays the number of selected papers each year on COVID-19-related fake news detection. COVID-19 was a novel research topic for everyone in 2020 since that was the beginning of the pandemic. The quantity of research has increased since 2020. Researchers continue to investigate this domain in 2024 to identify false news analysis on Twitter.

Among the comparative research works, the authors in [8] develop a system for identifying false information on Twitter. This system integrates ensemble learning with six distinct machine-learning algorithms. In addition, they employ an ensemble-stacking technique by combining the selected machine-learning models to enhance the overall performance of the model. This enhances the accuracy (97.8%) and overall applicability of the model. The authors mention the issue of small training dataset size because Twitter API does not allow the quick retrieval of Tweets. Furthermore, since the authors recommend the use of the stacking strategy, they also acknowledge that the model may encounter the issue of overfitting and higher prediction times. Subsequently, another effort [9] utilizes two machine learning (ML) supervised methods, namely Random Forest (RF) and Decision Tree (DT) classifiers, to identify fake news text data related to COVID-19. Although the accuracy is the same as the above-mentioned work, their prediction time is faster (1.25 times) this time since they utilize simple models instead of a stacking strategy. The work of [10] presents a model that automatically annotates the text in tweets by leveraging supporting assertions. Their ensemble strategy employs a fusion of various machine-learning models to identify fabricated news. The only problem with their model is that they have lost the interpretability. Another work [11] employs various state-of-the-art machine-learning techniques to classify the COVID-19 dataset. Regarding their work, the Decision Tree (DT) algorithm has superior accuracy (97.9%) compared to the Support Vector Machine (SVM) and Naïve Bayes (NB) algorithms. The authors mention the issue of the Bias-Variance trade-off and feature misinterpretation as their limitations.

Table 3 presents the summary information gathered from the relevant papers on COVID-19-related misinformation detection on Twitter.

**Table 3:** Summarized information from the COVID-19-related misinformation detection articles is presented here.

Title	Methods	Contribution	Author	Publisher	Citation
Detection of COVID-19 fake news text data using random forest and decision tree classifiers	RF, DT classifiers	Shows high accuracy and low prediction time.	[9]	IJCSIS	20
COVID-19 fake news detection model on social media data using machine learning techniques	SVM, NB, DT	Improves the accuracy.	[11]	IEEE	1
Detection of fake news text classification on COVID-19 using deep learning approaches	LSTM, BiLSTM, GRU, RNN, CNN, NB, SVM, KNN, RF, LR, DT, Adaboost, NN	Discusses that DL classifiers are highly proficient.	[12]	Hindawi Limited	83
Multichannel convolutional neural networks for detecting COVID-19 fake news	CNN	Helps the classifier identify false news more precisely by CNN model with three separate embedding channels.	[13]	Oxford University Press	3
Multi-Context based neural approach for COVID-19 fake news detection	MiCNA, a multi-context neural architecture	Improves performance by utilizing information from three pre-trained transformer-based models, BERT, BERTweet, COVID-Twitter-BERT.	[14]	ACM Digital Library	1
Fake or real news about COVID-19? Pretrained transformer model to detect potential misleading news	Fusion vector multiplication, Ensemble Transformer model, CT-BERT-RoBERTa	Demonstrates the ensemble DL architecture, superior performance compared to CT-BERT and RoBERTa by employing the multiplicative fusion technique.	[15]	Springer	25
COVID-19 fake news detection: A hybrid CNN-BiLSTM-AM model	Hybrid model, CNN-BiLSTM-AM	Proposes the generation-spread-identification-refutation framework for guiding public opinion in detecting emergency news effectively.	[16]	Elsevier	7
ANTi-Vax: a novel Twitter dataset for COVID-19 vaccine misinformation on detection	XGBoost, LSTM, BERT	Demonstrates a novel approach for detecting misinformation related to COVID-19 vaccines, using machine learning methods.	[17]	Elsevier	131
Using deep learning models to detect fake news about COVID-19	LSTM, GRU, BiLSTM	Compares misinformation detection using deep learning model among LSTM, GRU, and BiLSTM models. BiLSTM yields the best accuracy.	[18]	ACM	31
Towards COVID-19 fake news detection using transformer-based models	BERT, CT-BERT, NN structures	Achieves optimal performance by investing in innovative transfer learning approaches using transformer-based techniques with various downstream Neural Network architectures.	[19]	Elsevier	14
COVID-19 fake news prediction on social media data	Multinomial NB, LR, SVM, DistilBERT	Compares between traditional and distilBERT model. DistilBERT yields better accuracy.	[20]	IEEE	7
FakeBERT: Fake news detection in social media with a BERT-based deep learning approach	ALBERT	Uses bidirectional training techniques the BERT combined many simultaneous blocks of a deep CNN with varying kernel sizes and filters.	[21]	Springer	490
COVID-19 fake news detection using bidirectional encoder representations from transformers-based models	BERT	Adds BiLSTM and CNN layers with frozen or unfrozen parameters to BERT. Finds the most effective model is BERT fine-tuned model with frozen parameters, and BiLSTM layers perform best.	[22]	arXiv	14
Transfer learning and GRU-CRF augmentation for COVID-19 fake news detection	BERT & GPT2 as pre-trained using the BiGRU-Att-CapsuleNet model, BiGRU-CRF features augmentation	BiGRU-Att-CapsuleNet(BiGRU-CRF) model to compare the technique on a standard LSTM, Bi-GRU, BiGRU-Attention, and BiGRUAttention-Capsule. The hybrid model with augmentation achieves higher accuracy.	[23]	doiSerbia	17
Bilingual COVID-19 fake news detection based on LDA topic modeling and BERT transformer	LDA, BERT	Improves domain-specific case resolution by Adding topic information to BERT's pre-trained contextual representations.	[24]	IEEE	1
Machine learning-based identifications of COVID-19 fake news using biomedical information extraction	Biomedical information extraction techniques with ML	Shows the biological information impacts in ML models lay the foundation for computational COVID-19 fake news detection methods.	[25]	MDPI	6
Dynamic probabilistic graphical model for progressive fake news detection on social media platform	Dynamic probabilistic graphical model	Improves the Kalman filter to the labeled variable dimension Kalman filter(LVDKF), which is good for detection.	[26]	ACM	15
A multi-layer approach to disinformation detection in US and Italian news spreading on Twitter	Multi-layer and network-based approach	Quantifies the advantage of separating the layers over an aggregated approach and evaluate each layer's classification impact.	[27]	Springer	41
The performance of graph neural network in detecting fake news from social media feeds	GNN	Shows GNN-based models can perform better than baseline LSTM in terms of accuracy.	[28]	IEEE	4
Explainable text classification model for COVID-19 fake news detection	LIME-BiLSTM model	Assures BiLSTM classification accuracy, LIME ensures transparency and explainability of COVID-19 fake news classification, and the model becomes comprehensible.	[29]	JISIS	9
Combat COVID-19 infodemic using explainable natural language processing models	DistilBERT, SHAP (Shapley Additive Explanations)	Proposes an explainable natural language processing model to combat misinformation about COVID-19 due to their efficiency and effectiveness.	[30]	Elsevier	115
An efficient model for detecting COVID fake news using optimal lightweight convolutional random forest	Lightweight convolutional random forest-based honey badger (LCRF-HB)	Shows the most effective parameter values for the LCRF-HB result in enhanced performance (hyperparameter configuration).	[31]	Springer	0
Graph global attention network with memory: A deep learning approach for fake news detection	GANM	Utilizes three graph convolutional networks to extract significant characteristics from the news propagation network and combine internal and external user information.	[32]	Elsevier	2
Advancing fake news detection: hybrid deep learning with FastText and Explainable AI	LIME, LDA, CNN-LSTM layers with FastText embedding	Experiments with advanced transformer-based models, augmenting with hyperparameter adjustments, and it outperforms traditional RNN-based frameworks.	[33]	IEEE	2

One of the most recent studies conducted by the authors in [34] investigates the efficacy and dependability of employing Naïve Bayes algorithms to identify fake information regarding COVID-19 in social networks. The authors show that complement Naïve Bayes (CNB) is an effective tool for detecting online fake news, achieving the highest accuracy (98.9%) and the shortest runtime (100 milliseconds per prediction), however, their model is plagued by the independence assumption problem. Another recent work [35] investigates a new Environmental Uncertainty Perception (EUP) framework that incorporates the uncertainty of the information environment into misinformation features. The objective is to improve the accuracy of the model (98.91%) in tasks such as detecting misinformation and estimating the extent of its spread. The researchers have evaluated the efficacy of the EUP by analyzing real-world data sets of COVID-19 misinformation. Measuring the extent of misinformation spread is a new horizon in this research domain, despite the fact that the measure of the extent of misinformation is highly subjective and ambiguous, their model is afflicted by the independence assumption problem. Furthermore, the dataset is notably limited in size.

Deep learning-based algorithms are also being used to detect COVID-19-related fake news from the very early age of the pandemic. The experiment conducted by [12] demonstrates that their Deep Learning (DL) classifiers have a high level of proficiency (accuracy 95%, precision 93.33%, recall 95%, F1-score 94.02%) in appropriately identifying disinformation with a problem of smaller dataset size. The endeavors of [13] introduce a Convolutional Neural Network (CNN) model with three distinct embedding channels. This model aids the classifier in accurately (accuracy 98.31%, F1-score 97.02%) detecting fake news by providing contextualized text representation, embedding for static semantic terms, and embedding for lexical words. A loss of interpretability is an issue with their models. One of the notable observations is that there are a plethora of advanced DNN sequential and stacking models used in this fake news detection domain. For example, the effort of [36] presents a methodology that is designed to identify and combat the dissemination of fake information on COVID-19, with a specific focus on Twitter. The researchers employ a machine-learning (ML) model for detecting misinformation, particularly LSTM networks, which are a specialized sort of recurrent neural network (RNN). Additionally, they utilize a Multichannel Convolutional Neural Network (MC-CNN) and the k-nearest neighbors (KNN) algorithm. The authors achieve an accuracy of 97.33% and an F1-score of 97.11% but at the cost of higher prediction time (937 milliseconds per prediction). Another researcher [37] addresses the capacity of deep learning models, specifically CNN, to effectively identify false information in COVID-19-related tweets. Additional shortcomings with this model include a longer amount of time required for prediction and a limited dataset size.

Several recent studies have also investigated the use of hybrid deep neural network models to identify false information in social networks. The authors of [16] suggest a hybrid model named ‘CNN-BiLSTM-AM’ (Convolutional Neural Network (CNN), Bidirectional Long Short-term Memory Network (BiLSTM), and Attention Mechanism (AM) models can accurately detect COVID-19-related fake news. Their model achieves an accuracy of 98.7% but requires a large dataset and high computational cost. Another author [14] has introduced MiCNA, a multi-context neural architecture that surpasses both the baseline and candidate models (three transformer designs) and establishes itself as the leading COVID-19 fake news detection model. Although this model achieves an accuracy of 98.69%, it is susceptible to overfitting because the dataset size is comparatively small. The work of [18] utilizes other Deep Learning techniques, including LSTM, GRU, and BiLSTM models, to detect and classify bogus news. Out of these options, BiLSTM exhibits the highest accuracy (98.9%). This model has an issue with vanishing gradients. The recent work of [15] utilizes fusion vector multiplication to enhance the model's ability to identify potential instances of false news. The researchers in [28] compare the effectiveness of a GNN-based model for detecting fake news on social media threads to a classic sequential model, LSTM. This study shows that, in terms of precision metric (98%), GNN-based models perform better than baseline LSTM. Oversmoothing is a problem with this model. The authors in [38] have presented a new probabilistic fusion technique to merge the knowledge obtained from two language models, BERT-CNN and BERT-LSTM. When different parameter values are used, the detection accuracy (99%) surpasses that of the current approaches for detecting fake news. The authors suggest a fusion technique that utilizes the Bayesian theorem for score-level fusion to enhance the performance of false news detection. They compare their method with BERT-LSTM and BERT-CNN, which serve as the baseline models. Consequently, they have concluded that their hybrid model outperforms other models that solely utilize BERT, word2vec, or BoW techniques. Due to the utilization of the embedding layer and BERT network, the computational cost of the model is high. Additionally, the model is afflicted by the issue of vanishing gradients and the assumption of independence.

A few initiatives have been taken to utilize the unsupervised methods in this scope. For instance, in a very recent study, the authors [39] propose GAMC, an unsupervised method for detecting fake news. GAMC utilizes the Graph autoencoder with masking and contrastive learning. Their strategy utilizes both the context and content of news propagation as self-supervised signals to decrease reliance on labeled datasets. Afterward, a mechanism for encoding and decoding graphs has been applied as well and their approach demonstrates significant efficacy in identifying fake news. One important thing to mention here is that while the sequential and stacking models yield superior accuracy, they experience a well-known issue called the Vanishing Gradient problem, especially when the length of the content in the tweet is excessively long. Observing this issue, the authors of [40], in their study, present a comparison of artificial neural networks, demonstrating that the simple Artificial Neural Network (ANN) achieved superior performance compared to more complex deep Learning methods, such as Convolutional Neural Networks (CNNs) and Recurrent Neural Networks (RNNs). Furthermore, the amalgamation of the datasets has led to enhanced performance as compared to the separate datasets. Regarding the duration of execution, the Artificial Neural Network (ANN) demonstrated superior performance by exhibiting a lower training time. Other undertaking [25] employs biological information extraction (BioIE) and machine learning techniques to forecast the dissemination of false information on COVID-19. The incorporation of BioIE-based features enhances the performance of a cutting-edge multi-modality model. Similarly, the researchers [41] demonstrate that graph link prediction surpasses categorization in the context of misinformation detection.

Among other most recent research works for fake news detection, the article by [32] presents a Graph Global Attention Network with Memory (GANM). It utilizes three graph convolutional networks to extract significant characteristics from the news propagation network and combines internal and external user information. The suggested method uses deep learning, graph neural networks, and temporal modeling to detect fake news in complicated graph-structured data. The amalgamation of multiple models yields better performance (99.05%) but at the cost of a higher prediction time (999 milliseconds) and over-smoothing issues. To achieve lower prediction times, a new idea of using probabilistic and filter-based approaches has also been introduced. A group of researchers [26] suggest a Dynamic Probabilistic Graphical Model for Social Media Progressive Fake News Detection. After observing real-world datasets, they adaptively improve the Kalman Filter to the Labeled Variable Dimension Kalman Filter (LVDKF), which learns two universal patterns from true and fake news to capture unevenly arriving time-series data. Progressive detection can be achieved by taking sequential data, distilling post-dynamic evolution information, and using crowd wisdom from user answers. After deriving the formulas using the Forward, Backward, and EM algorithms, they have constructed a Bayes' theorem-based dynamic detection algorithm.

**Figure 4:**
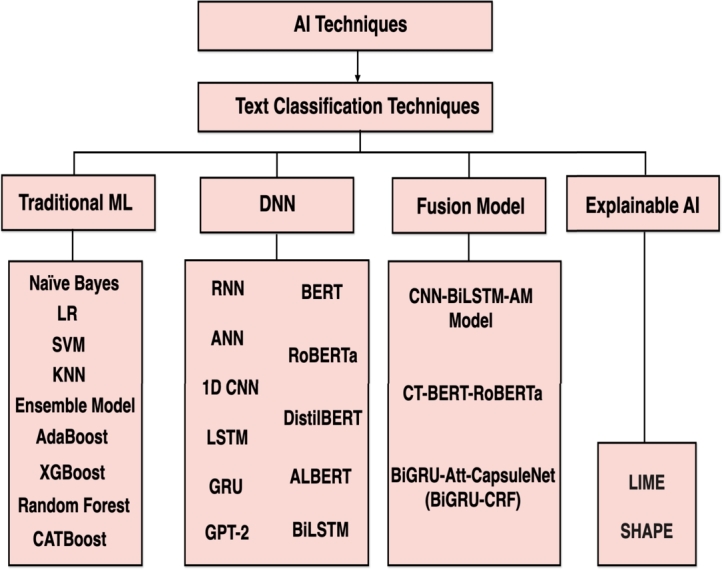
AI approaches used in different selected literature are summarized in this plot.

Since different types of models have been proposed to achieve the sole goal of detecting fake news, we try our best to tally the models being used in this scope. Fig. 4 shows that various AI approaches were employed in literature on a frequent basis. This gives the reader a high-level overview of what algorithms have been used so far and what else can be done within the scope. With the advent of transfer-based models, we observe a paradigm shift in this domain. A lot of researchers are now utilizing models, like BERT, that take advantage of attention mechanisms and embedding approaches. While we can achieve better accuracy, these attention transformers and embedding-based approaches suffer from higher prediction time as well as they need large training datasets to avoid overfitting. The researcher of [19] examines transformer-based models for detecting misinformation related to COVID-19 and devises novel and enhanced methodologies for this purpose. These novel approaches employ transformer-based models to enhance the comprehension of COVID-19 misinformation beyond the capabilities of both conventional and sophisticated machine-learning techniques. They attain a 99% accuracy rate at the expense of increased prediction time and interpretability loss. One of the notable tasks of [20] conducts a comparison between a traditional ML-based model and a DistilBERT model. Transformer-based models are excessively reliant on data and computational resources due to some limitations on memory, time, and energy consumption. In this instance, a group of researchers has discovered a novel, semi-supervised technique for detecting fake news that is both efficient and productive. They have achieved this by utilizing a content-oriented classifier that relies on a compact BERT embedder. Their method achieves good detection performance with few training samples, low human participation, and compute/memory costs [42]. In their study, the deep learning-based approach outperformed (98.8% accuracy) the traditional ML models. The effort of [21] employs an ALBERT-based (Bidirectional Encoder Representations from Transformers) deep learning methodology with an accuracy of 98.90% and a prediction time of 1 seconds, while the work of [43] utilizes the pre-trained BERT and RoBERTa models with lower accuracy 98% and prediction time 902 milliseconds for the identification of COVID-19-associated misinformation. The study of [22] utilizes a pre-trained Bidirectional Encoder Representations from Transformers (BERT) model, along with BiLSTM and CNN layers, to fine-tune the BERT model using either frozen or unfrozen parameters. Their suggested model demonstrates superior performance in detecting bogus news related to COVID-19 with an accuracy of 98.99% but their training dataset size is also very small. To find the optimal accuracy for COVID-19 misinformation detection, the authors of [44] apply deep learning methods utilizing BERT, LSTM, and BLSTM architectures to assess the effectiveness of three vectorization techniques: Bag of Words, Word2Vec, and BERT embedding. Their empirical findings demonstrate that the LSTM model with BERT yields the most optimal performance. The embedding approaches come with the advantage of better feature representation and improved performance but at the cost of extra processing time. They achieve the highest performance (accuracy 99.1%, F1-score 98.9%) but with a cost of prediction time of 1.1 seconds due to extra embedding time. Many more efforts have been undertaken to take advantage of GPT and Natural Language Processing (NLP) in this domain, which we also opt to summarize. The recent task of [23] introduces a method called transfer learning and GRU-CRF augmentation to detect fake news related to COVID-19. Essentially, the model is a combination of BERT and GPT2, which have been pre-trained using the BiGRU-Att-CapsuleNet model and BiGRU-CRF feature augmentation. All models are pre-trained using BERT and OpenAI GPT2. BERT consistently shows superior performance compared to GPT2 in all models. Other works, like [24] propose a bilingual model using Latent Dirichlet Allocation (LDA) topic modeling and the BERT transformer to detect COVID-19 fake news in Persian and English with good accuracy (92.18%). This shows that adding topic information to the BERT network's pre-trained contextual representations improves domain-specific instance solving. The author Pierri et al. [27] proposes a multi-layer representation of Twitter diffusion networks where each layer describes one type of interaction (tweet, retweet, mention, etc.), while [41] demonstrates that graph link prediction surpasses categorization in the context of misinformation detection.

Another author [45] introduces a categorization methodology that utilizes novel characteristics of tweets, employing techniques such as natural language processing, machine learning, and deep learning. The method is executed concurrently using the Apache Spark framework. Empirical evidence demonstrates that employing this method in conjunction with the random forest algorithm produces highly beneficial outcomes. Furthermore, they illustrate the significance of sentiment analysis in the identification of false information. The latest endeavors of [46] propose an automated framework, FNEC (Fake News Encoder Classifier). This framework used the transformer-based model ELECTRA (Efficiency Learning an Encoder that Classifies Token Replacements Accurately) to enhance accuracy. In order to assess its effectiveness, the suggested approach FNEC was compared to several state-of-the-art techniques (namely, SVM, NV, PAC, LSTM, Bi-LSTM, and BERT) using standard performance metrics. To ascertain the most optimal approach, the recent study of [47] conducts experiments using several machine learning (ML) and deep learning (DL) approaches to assess their performance on the dataset, including fake news in recent works. The researchers employ a range of advanced NLP-based embedding techniques, including TF-IDF, n-gram, Word2Vec, and GloVe, to identify the most effective combination that boosts the detection process and enhances the accuracy of the classifier. Finally, it is noted that deep learning models, specifically LSTM and CNN-LSTM, exhibited superior performance in comparison to conventional machine learning models. Even though, they mention the chance of overfitting and high prediction time, which we observe as a general problem in most of the DNN-based models.

Explainable AI is a recent research focus within the realm of contemporary deep learning. Several studies aim to enhance the reliability of AI text categorization systems by evaluating the performance of machine learning classifiers. The work of [48] states that both global and local explanations are provided to help users understand the model's behavior, promoting transparency and building trust among AI users. A model proposed by [29] is an integrated LIME-BiLSTM model, wherein BiLSTM guarantees the accuracy of classification while LIME ensures the transparency and explainability of the COVID-19 fake news classification. For the purpose of combating disinformation regarding COVID-19, the authors [30] suggest an explainable natural language processing model that is based on DistilBERT and SHAP (Shapley Additive Explanations). This model is characterized by its efficiency and effectiveness. We conclude that less work is being done utilizing this kind of model, mostly because it is still a new research field.

Recently, research has focused on optimizing the performance of machine learning (ML) and deep learning (DL) models by preventing overfitting and providing robust, generalizable outcomes. For instance, strategies like regularization methods, optimization techniques, hyperparameter tweaking, and FastText embedding have been used for enhanced detection and model generalization to address the overfitting issue [33]. Their suggested hybrid model, which integrates CNN and LSTM layers with FastText embedding, surpasses existing models accurately. Subsequently, they acquire a more profound understanding of the model's decision-making process by employing explainable AI techniques such as LIME and LDA. In addition, they have used advanced transformer-based models such as BERT, XLNet, and RoBERTa, augmenting them with hyperparameter adjustments. Transformer models outperform RNN-based frameworks in syntactic nuances, helping semantic interpretation. To summarize, traditional ML models yield lower prediction time while they suffer from lower performance. DNN-based models exhibit superior performance, albeit being susceptible to issues such as vanishing gradients, higher prediction times, and overfitting. Table 4 demonstrates this scenario. It compares the performance between traditional and deep learning-based top-performer models. Models optimization techniques, like utilizing pre-trained models, regularization, etc., have been undertaken but there is still an issue with overfitting due to the fact that the training dataset is too small in all of these research works. We think increasing the training dataset size can be a good research direction. We elaborate on the dataset being used in this scope later in this paper.

**Table 4:** Comparison between high performer traditional and deep learning based models to predict fake news.

Type	Cite	Main Model	Accuracy	F1-Score	Limitation
Traditional	[8]	Ensamble-Stacking	97.8	97.3	Small dataset, Overfitting
Traditional	[11]	DT	97.9	97	Bias-Variance trade-off
DNN	[16]	CNN	98.7	98.5	Overfitting
DNN	[38]	BiLSTM	99	98.8	Vanishing Gradient
DNN	[44]	BERT	99.1	98.9	High computation time, Overfitting

## 4 COVID-19 misinformation disseminators analysis on Twitter

Prominent researchers not only predict fake news but also publish plenty of research studies on COVID-19-related fake news ‘analysis’ regularly. These works include mostly the impacts of fake news on public health or fake news detection techniques. Three researchers from the Massachusetts Institute of Technology have analyzed each verified true and deceptive story disseminated on Twitter. According to [49], the dissemination of disinformation in the online environment is more extensive than that of real data. Therefore, many research studies have focused on analyzing the activities of the disseminators over the years. We try our best to gather the papers from each year and construct a comparative study here.

**Figure 5:**
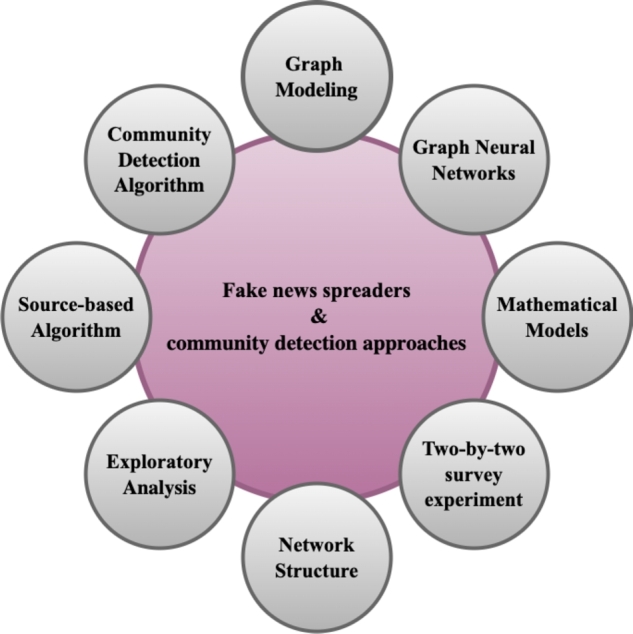
Methods/Algorithms used to identify the fake news spreaders and detect their communities in various papers are summarized here.

**Figure 6:**
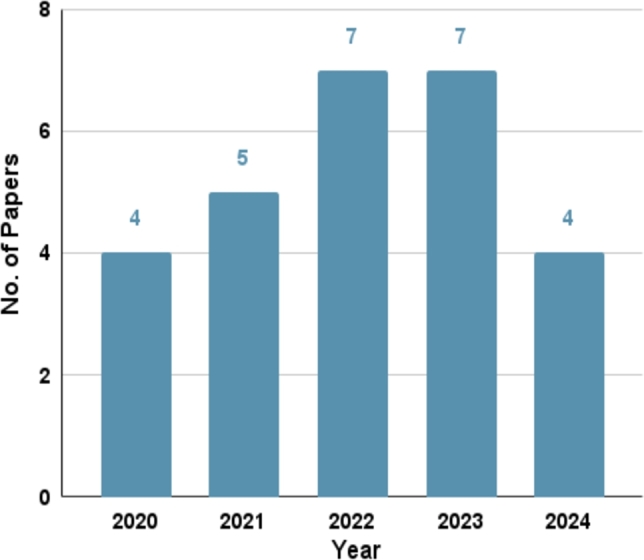
Yearly paper analysis of the disseminator and their community of COVID-19 misinformation on Twitter.

Fig. 6 demonstrates the number of papers on disseminator analysis selected from each year. These papers have proposed different methods and techniques to analyze the spreader's activities. The techniques being used in analyzing the dissemination of fake information and the identification of their communities, as described in the majority of academic papers, are outlined in Fig. 5. We observe that a plethora of methods, for example, graph-based analysis, community analysis, exploratory analysis, etc., have been proposed to investigate fake news spreaders.

The very first initiative [50] introduces a framework for detecting the spread of fake news that is the first of its kind to utilize network structure and historical behavioral data rather than content. Besides that, they employ inductive representation learning to identify and combine trust-based features from weighted network node neighborhoods. However, their inductive representation learning model is plagued by (i) loss of interpretability, and (ii) lack of generalization to unseen data. Later, another effort [51] demonstrates a spatial and temporal correlation between the transmission of information and the occurrence of new COVID-19 cases. Furthermore, they have found that misinformation and low-quality information are less prevalent compared to other themes specific to the crisis. This investigation aims to examine the discussion surrounding COVID-19 on social media. But still, their work is hindered by the issue that inconsistent data collection and reporting standards in different regions could lead to in inadequate or erroneous datasets, hence confounding research. A contemporary effort in [52] analyzes COVID-19 misinformation communities by comparing their network structure, sociolinguistic variation, and membership in disinformation campaigns with other health-related misinformation communities. It is suggested that misinformation networks are more complicated due to their organization and analysis. This work also has not considered the dynamic nature of the user behavior. Similar to this community detection technique, [53] focuses on identifying important users from Twitter data using the TwitterRank algorithm and suggests a community detection algorithm. Tweets on COVID-19 and 5G conspiracy ideas have also been investigated to find misinformation spreaders. Still, this work has not considered (i) data sparsity, (ii) temporal dynamics, and (iii) computational complexities, which pose as their major limitations.

In 2021, a FNSC (Fake News Spreader Classifier) is proposed by [54]. They use a computational approach to extract features from the social media posts of these users to recognize who is a fake news spreader for a given topic. This model is topic-sensitive, meaning that accurately categorizing tweets into topics can be challenging and may lead to misclassification, affecting the reliability of the influence. Other researchers [55] use Louvain community detection algorithms on graphs (graph modeling) to analyze social networks and evaluate fake and true news dissemination. This work is highly dependent on parameters like ‘Resolution limit’ and may encounter the problem of local optima. The authors in [56] develop a source-based algorithm to detect content from news propagators, such as posters and re-tweeters. They have compared the proposed methodology to a real-world COVID-19 dataset using machine learning and deep learning models at community and node levels. Despite this, their technique is unable to detect hidden or latent sources and faces challenges in terms of scalability. Another study [57] suggests using complex networks and machine learning techniques to improve Twitter hacker detection. Their network Cyber-Twitter model can detect hackers and warn of prospective attacks on future institutions and individuals. Nonetheless, the definition of hackers can be highly subjective which pose as the limitation of this work. According to the authors from [58], a Graph Transformer Network (GTN) can learn efficient node representations and identify significant relationships between nodes in the original network to detect disinformation. Basically, it shows propagation-based fake news detection using graph neural networks with transformers. This work drawbacks from (i) scalability, (ii) sparsity, and (iii) over-smoothing issues. Another endeavor [59] finds that fake news elements in messages with incorrect content are circulated and adopted as pristine communications and social prestige does not influence maladaptive trait spread. Due to the minimal cultural exchange fostered by social network personalization and population culture, Twitter information on fitness may not be credible.

In 2022, the researchers in [60] conduct a study to gain insights into and describe influential sources of misinformation. They also conducted an exploratory analysis of the Twitter activity of these sources, comparing their online behavior with that of a group of users who actively shared accurate and useful information during the COVID-19 pandemic. They have considered the dynamic behavior of the users though. There are two categories of fake news spreaders: 14% active and motivated authors and 86% consumers who prefer to repeat others' information without creating their own, as explained by [61]. The researchers combine structural network data, including node in-degree, with metadata about retweets' contents, which effectively distinguishes fake news producers from retweeters, regardless of the threshold chosen by them. The study conducted by [62] proposes a two-by-two survey experiment and finds that misinformation propagates very quickly and Twitter users trust fake information ties with more likes, comments, and retweets than celebrity news without any proof. Another study by the same authors [63] conducts a comparative evaluation of state-of-the-art models using a corpus linguistics approach. They specifically focus on recent Transformer-based architectures to detect FNS (fake news spreaders). Furthermore, the researchers have discovered the most effective model (a shallow CNN) for identifying FNS in the dataset by hyper-parameter tuning. Their study faces the issue of the presence of biases in data and scalability. Further study [64] introduces the HC-COVID, a hierarchical crowdsource knowledge graph-based framework, as a solution to the challenge of detecting explainable COVID-19 misinformation on social media. The researchers suggest a new approach called a dual hierarchy attention-based graph neural network for HC-COVID. This method aims to identify and clarify misinformation related to COVID-19 by analyzing both specific and generalized knowledge facts obtained from the constructed graph. The experimental findings unequivocally show that HC-COVID is highly effective in detecting misinformation explanations related to COVID-19. However, this work has the following limitations: (i) validation and verification issues and (ii) bias and representativeness issues. In 2023, a group of researchers utilize an interdisciplinary approach to identify and characterize tweets containing COVID-19 misinformation [65], [66]. Then, the authors of [67] use attribute assortativity, which helps to understand node connections by attributes, and Graph Neural Networks (GNNs) for node label classification to analyze Twitter misinformation spreaders' probability. Assortativity levels can be used to determine how different the network connections are between disseminators of misinformation and those who do not. Subsequently, Graph Neural Networks (GNNs) are employed to categorize individuals who disseminate misinformation by effectively capturing complex relationships and dissemination patterns between nodes through the utilization of structural information within the network. This technique considers network structure, network properties, and interactions between nodes, yielding a thorough understanding of the variables influencing the dissemination of false information. The limitations of this work are (i) gradient vanishing and exploding issues, (ii) scalability, and (iii) over-smoothing issues.

The authors of [68] present a novel method for detecting potential anomalous nodes that disseminate misinformation on Twitter networks. They utilize Graph Neural Networks (GNNs) and entropy-based techniques to identify these anomalous nodes. By combining node embeddings with entropy-based methodologies using GNNs, this methodology shows promise in understanding the behavior of different types of misinformation propagators. Although this approach is plagued by two main problems: graph isomorphism and interpretability issues. Another study is carried out [69] on US physicians to understand COVID-19-related misinformation propagated by them on social media and their characteristics. They further advocate for meticulous examination of harm caused by physicians, who are uniquely trusted to spread misinformation, and for ethical and legal rules for misinformation dissemination.

Although it is early 2024, several papers have already been published on fake news spreader analysis. For example, the authors in [70] propose a Word2Vec model, a topic lexicon, multiple regression models for topic diversity, and conspiracy theories to shape engagement with COVID-19 misinformation on Twitter. This work contains the following limitations: (i) topic sensitivity, and (ii) computational complexity issues.

Table 5 summarizes the research works of misinformation disseminator investigation and their community analysis related to COVID-19 data on Twitter. Just by having a look at Table 5, interested readers can have a view of the works being done in this scope. In the subsequent paragraphs, we provide further details and explanations on the works indicated in the table.

**Table 5:** Misinformation disseminator investigation and their community analysis related articles' summaries are presented.

Title	Methods	Contribution	Author	Publisher	Citation
Detecting fake news spreaders in social networks using inductive representation learning	Graph neural network, Inductive representation learning	Proposes a graph neural network-based approach to identify nodes that mostly become spreaders and an inductive representation learning framework to predict nodes in a densely connected community.	[50]	IEEE	21
Automated classification of fake news spreaders to break the misinformation chain	Linguistic model, FNSC, computational approach to extract features	Shows FNSC classifier turns user posts into a high-dimensional feature matrix, that is a transformer-based DNN architecture used to classify users.	[54]	MDPI	23
Interdisciplinary approach to identify and characterize COVID-19 misinformation on Twitter: Mixed methods study	Interdisciplinary approach	Shows an interdisciplinary team combined computational and qualitative methods to gain a better understanding of COVID-19 misinformation.	[65]	JMIR	0
The voice of few, the opinions of many: evidence of social biases in Twitter COVID-19 fake news sharing	Dynamic modeling, convergent cross-mapping	Shows that fake news spreaders are divided into ‘creators’ and ‘consumers’. A small percentage of individuals are responsible for the most inaccurate information on Twitter.	[61]	Royal Society	14
Fake news analysis and graph classification on a COVID-19 Twitter dataset	Graph modeling	Discusses social network analysis and compares the characteristics using community detection algorithms on the graphs.	[55]	IEEE	6
Complex network and source-inspired COVID-19 fake news classification on Twitter	Source-based algorithm, hybrids outperform network, and user features	Use the source-based method to detect community and node-level ML and DL models. Ensemble's CATBoost and RNN DL models perform well.	[56]	IEEE	38
Communication of COVID-19 misinformation on social media by physicians in the US	MiCNA, a multi-context neural architecture	Discusses COVID-19 misinformation types propagated by US physicians after vaccines and the characteristics of the physicians spreading misinformation.	[69]	JAMA network	23
Constructing a user-centered fake news detection model by using classification algorithms in machine learning techniques	XGBoost, SVM, RF, LR, CART, NNET	Uses ML classification models and compared fake news detection rates to indicate what makes fake news propagate. RF predicted most correctly, whereas NNET performed lowest.	[66]	IEEE	4
The disinformation dozen: an exploratory analysis of COVID-19 disinformation proliferation on Twitter	Exploratory analysis	Analyzes the understanding and characterization of prominent misinformation influencers.	[60]	ACM Digital Library	39
Cultural evolution and digital media: diffusion of fake news about COVID-19 on Twitter	Predictive model, ANN, MLP	Compares using numerous deep learning models for fake news identification, among LSTM, GRU, and BiLSTM models BiLSTM detects best with accuracy.	[59]	Springer	19
A first look at COVID-19 information and misinformation shared on Twitter	ALBERT	Shows a technique for bidirectional training that uses the BERT combined with many simultaneous blocks of a deep CNN with varying kernel sizes and filters.	[51]	arXiv	354
Efficient detection of hacker community based on Twitter data using complex networks and machine learning algorithm	Cyber-Twitter model	Proposes hacker efficiency detection using the complex networks technique with adapted ML algorithms.	[57]	Journal of Intelligent & Fuzzy Systems	8
Lexicon-based sentiment analysis to detect opinions and attitudes towards COVID-19 vaccines on Twitter in Italy	ML, LSTM, MC-CNN, KNN	Focuses on specific events of the vaccination campaign. The results highlighted an overall negative sentiment, especially for common users. The result shows different attitudes of opinion holders towards specific key events.	[71]	Elsevier	14
Propagation-based fake news detection using graph neural networks with transformer	Multinomial NB, LR, SVM, DistilBERT	Compares both traditional and distilBERT model, and DistilBERT gives good accuracy.	[58]	IEEE	14
Topic diversity and conspiracy theories shape engagement with COVID-19 misinformation on X/Twitter	Word2Vec model, topic lexicon, multiple regression models	Finds COVID-19 misinformation conspiracy stories have a high topic variety. The incorporation of conspiracy theories and increasing topic diversity on X/Twitter leads to increased social interaction (retweets, likes, and replies) with misinformation.	[70]	arXiv	0
Twitter and endorsed (fake) news: the influence of endorsement by strong ties, celebrities, and a user majority on the credibility of fake news during the COVID-19 pandemic	Two-by-two survey experiment	Discusses why and how misinformation propagates and Twitter users will trust fake information retweeted by a strong tie with more likes, comments, and retweets than celebrity news without any proof.	[62]	IJoC	11
Characterizing COVID-19 misinformation communities using a novel Twitter dataset	Network structure, linguistic patterns (LIWC)	Analyzes COVID-19 misinformation communities by comparing the network structure, sociolinguistic variation, and membership in disinformation campaigns with other health-related misinformation communities.	[52]	arXiv	185
Fake news detection in social media using graph neural networks and NLP techniques: A COVID-19 use-case	TwitterRank algorithm	Focuses on identifying important users from Twitter data using the TwitterRank algorithm and suggest a community detection algorithm.	[53]	arXiv	44
Epidemiological modeling of health information dynamics on Twitter	SEIR framework	Analyze the propagation of health information on Twitter using two mathematical models named TwitHComm, TwitHCommS	[72]	dlsu	0
Using GNNs for misinformation spreader detection via assortativity-aware node label classification in Twitter networks	GNNs, attribute assortativity	Shows complex relationships and dissemination patterns between nodes by using proposed techniques.	[67]	IEEE	0
Fake news spreaders detection: Sometimes attention is not all you need	SotA and non-deep SotA models, linguistics approach	Discover the most effective model (a shallow CNN) for identifying fake news spreaders.	[63]	MDPI	24
HC-COVID: A hierarchical crowdsource knowledge graph approach to explainable COVID-19 misinformation detection	HC-COVID, a hierarchical crowdsource knowledge graph-based framework	Shows experimental findings HC-COVID is highly effective in detecting and misinformation explanation related to COVID-19	[64]	ACM	40

In a recent paper, the authors [72] propose another non-ML/DL approach. They analyze the propagation of health information on Twitter using two mathematical models: the TwitHComm (Twitter Health Communication Model without sentiment) and the TwitHCommS (Twitter Health Communication Model with positive and negative sentiment), both based on the SEIR framework. These models offer useful insights into the dynamics of information dissemination and can serve as an example for developing methods and policies to control the distribution of information, particularly misleading and inaccurate information. The face the issues of (i) ambiguity and subjectivity, and (ii) polysemy.

With PageRank and other popularity and prestige metrics, the effort by [73] reveals COVID-19-related misinformation propagation patterns on the Twitter dataset in a recent work. Their community discovery module utilizes the graph outputs generated by the Network Creation module in order to identify and analyze cohesive communities. This module uses neighborhood information and community detection algorithms like the Girvan-Newman and Louvain algorithms to explain the organizational structures that disseminate false information. This methodology elucidates the methods by which misinformation proliferates within and among communities while also offering techniques for mitigating its impact. Furthermore, they have prioritized centrality metrics, reaction time, and reaction count as crucial indicators of an account's network importance in spreading misinformation. Apart from that, their work has the following constraints: (i) vulnerability to link manipulation, (ii) dynamic representation of authority, (iii) local minima, and (iv) computational cost issues. One of the recent studies on the dynamics of misinformation related to COVID-19 on Twitter utilizes text classification and BERTopic modeling to detect and categorize tweets containing misinformation [74]. The Leiden algorithm is employed in order to detect communities. The research indicates that influential and diverse societies disseminate misinformation to niche groups. The findings present significant insights into how online groups influence public discourse during crises by strategically disseminating misinformation. Nonetheless, this approach is hindered by its reliance on modularity optimization and its susceptibility to initialization sensitivity issues. One final comment to note is that most of the above-mentioned works are exploratory analyses and utilize existing algorithms to identify dominant spreaders, their characteristics, and their communities. Also, ranking the spreaders is totally subjective. Because of these reasons, the authors have not provided any hard evaluation metrics that we can use to compare one against another.

Finally, most of the research works have first constructed a network/graph-like architecture and, then, utilize that network/graph to extract dominant misinformation spreaders and/or their communities. There are various reasons why the researchers have preferred the graph architecture. First, graph architecture exhibits natural modeling and intuitive interpretation. Second, it preserves structural insights and identifies substructures. Third, there has been significant research and development in developing scalable algorithms for network community detection, making it feasible to analyze large-scale networks with millions of nodes and edges via parallel processing. Now, utilizing the graph architecture, popular dominant spreader extraction methods are TwitterRank and PageRank because finding dominant nodes is very straightforward using these methods. The researchers have mainly used Girvan-Newman and Louvain algorithms to extract communities from the graph architecture. Although these community-finding algorithms are quadratic in nature, the reasons for choosing these algorithms over others are that (i) the theory is very much intuitive and any variation of these algorithms is easy to incorporate, (ii) there are well-known Python packages for these algorithms and they are easy to implement as well, and (iii) these algorithms provide more accurate and simplified output than others. Nevertheless, we still feel that insufficient work has been done within this scope. For example, none of the works dig deeper into the communities they find. We suggest some more of the future research directions in the Discussion section later.

## 5 COVID-19 related dataset on Twitter

This section covers the examination of an extensive dataset comprising tweets pertaining to COVID-19, as well as the discussion of data pre-processing techniques and technologies.

The accomplishment of [75] examines a preliminary fact-check dataset obtained from a multilingual cross-domain, publicly available COVID-19 dataset gathered from 105 countries. Natural Language Processing (NLP) techniques are used to pre-process the data and manually categorize the fact-checked articles into different categories. This process involves meticulously cleaning up the data by deleting invalid URLs and duplicate entries. The article in [76] describes another large multilingual dataset of over 123 million tweets that was collected through the Twitter API. The authors of [77] have developed an intriguing approach to address the challenges related to the spread of misinformation during the COVID-19 pandemic. This model enables a comprehensive examination of data that is spread across various platforms and contexts. More precisely, it can concentrate on five distinct social media platforms and analyze user involvement regarding COVID-19. The effort of [8] proposes an ensemble learning framework that aims to verify the integrity of a vast quantity of data that is assessed and labeled by human annotators. The pre-processing stage involves utilizing the raw data collected by the streaming API, evaluating and comparing performance, normalizing the data, and extracting relevant features.

In order to have a deeper comprehension of the attention patterns exhibited on Twitter during COVID-19, we have shown a summarization of the COVID-19-related datasets and a characteristics analysis of fake news in Table 6. We have also built a database of datasets by compiling the training datasets from 17 different research works. The fellow enthusiasts can visit *https://github.com/AsmaUlHussna/COVID-19_dataset/tree/milestone* to gain access to our database, merge those datasets, and use that merged dataset to train their respective ML models. Some more fake news detection related training datasets can be found in the research works of [78], [79], [80], [81], [5], [20], [82], [83], [84], [85], [86].

**Table 6:** The COVID-19-related fake news investigation involves the utilization of datasets and their corresponding characteristics.

Dataset	Title	Pre-processing	Method	Author
Twitter dataset (tweets collected: 980,100)	Lies kill, facts save: detecting COVID-19 misinformation in Twitter	Standard NLP techniques.	C4.5, SVM, KNN	[8]
Twitter dataset (tweets collected: 72,922,211)	Machine learning to detect self-reporting of symptoms, testing access, and recovery associated with COVID-19 on Twitter: retrospective big data infoveillance study	Sampling, tokenization, remove hashtags, stop words, data cleaning, clusters, duplicate tweets.	Biterm Topic Model (BTM)	[87]
Twitter dataset (tweets collected: 4,196,020)	Twitter discussions and emotions about the COVID-19 pandemic: Machine learning approach	Remove hashtag symbol, non-English characters, special characters, punctuation, stop-words, analyze unstructured text data.	LDA	[88]
Twitter dataset	Conversations and medical news frames on Twitter: infodemiological study on COVID-19 in South Korea	Standard NLP techniques.	NodeXL, Clauset– Newman– Moore cluster algorithm	[89]
Twitter dataset	Risk communication in Asian countries: COVID-19 discourse on Twitter	Tokenization, filtering unnecessary textual information	LDA, PPL	[90]
TweetsCOV19	TweetsCOV19 - A knowledge Base of semantically annotated tweets about the COVID-19 pandemic	Harvesting, filtering, cleaning, semantic annotation, and metadata extraction.	RDF, CNN	[91]
Twitter dataset	Experts and authorities receive disproportionate attention on Twitter during the COVID-19 crisis	Normalizing texts, replaced account names, URLs, emails, removed emojis, fast text skip-gram model for 5 Epochs, context window size of 5, n-gram size between 3 and 6.	CAP, BERT	[92]
Twitter data	An exploratory study of COVID-19 misinformation on Twitter	NLTK, emoji package, URL removal using regular expressions.	LIWC, KLIP	[82]
Twitter data (tweets collected: 2,787,247)	Top concerns of Twitter during the COVID-19 pandemic: infoveillance study	Remove non-English tweets, retweets, punctuation, stop word, and non-printable character, normalizing Twitter users mention, lemmatizing texts.	LDA	[93]
Twitter dataset (tweets collected: 100,000)	Characterizing information leaders in Twitter during the COVID-19 pandemic	Content-based filtering, utilizing two concurrent filters for the streaming, removing poorly connected nodes.	DL	[94]
Twitter data (tweets collected: 67.4 million)	Disinformation and misinformation on Twitter during the novel coronavirus outbreak	Standard NLP techniques.	ML	[95]
Twitter dataset (tweets collected: 6,667)	COVIDHealth: A benchmark Twitter dataset and machine learning-based web application for classifying COVID-19 discussions	Remove mentions, hashtags, URLs, repeated characters, punctuation, stopwords, and non-English languages.	NLTK, ML	[96]
Twitter data	MANIFESTO: a human-centric explainable approach for Fake news spreaders detection	Remove links, usernames, punctuations, Twitter special characters, contractions, lowercase words, and stopwords.	NN, classical classifiers	[97]

Another work of [87] demonstrates a high level of accuracy in distinguishing reliable and unreliable tweets, including COVID-19 content. A total of 4,492,954 tweets associated with COVID-19 symptoms were gathered. The analysis employs an unsupervised machine learning technique called the Biterm Theme model (BTM) [87]. This method groups tweets into clusters based on common themes that are associated with certain words. The pre-processing phase involves several steps, including sampling, tokenization, removal of hashtags, elimination of stop words, data cleaning, clustering, and removal of duplicate tweets. In another recent development [88], the authors utilize the Twitter dataset that was gathered from the Twitter public API between the dates of March 3 and 20, 2020. The researchers utilize an LDA model, employing a machine learning technique, to identify commonly appearing individual words, word pairings, important concepts, themes, and expressed ideas within the gathered tweets, which total more than 4 million tweets. The data pre-processing stage includes removing the hashtag symbol, excluding non-English letters, eliminating special characters and punctuation, and erasing stopwords to analyze unstructured text data. The researchers in [89] implement multi-coder techniques to identify and classify false information. The Clauset-Newman-Moore cluster algorithm with the Harel-Koren Fast Multiscale layout algorithm has been applied to analyze a dataset from Twitter with 43,832 Twitter users and 78,233 relationships. The pre-processing involves identifying multidimensional communication activity, conducting network analysis, and performing content analysis. Also, they suggest, in another paper, an automated approach for detecting and analyzing historical phase transitions in significant matters across these nations [90]. Pre-processing involves tokenizing the data into its smallest components and filtering out extraneous textual information using Python tokenizer packages specific to each language. Their Twitter dataset is analyzed using LDA and PPL.

The process of extracting the TweetsCOV19 subset from the TweetKB dataset has been explained by authors in [91]. They collect internet conversations about various aspects of the COVID-19 pandemic and its impact on society. TweetsCOV19 is a publicly available dataset containing over 8 million tweets. The pre-processing stage involves employing a parallelized annotation pipeline to eliminate spam using a multinomial Naïve Bayes classifier, does sentiment analysis, gathers pertinent data, eliminate extraneous information, sanitizes the data, label connotations, and eliminates metadata. Another researcher builds a Twitter dataset and develops a classifier using BERT [92]. The pre-processing stage involves normalizing texts, substituting account names, URLs, and emails, eliminating emojis, training a rapid text skip-gram model for 5 epochs, using a context window size of 5, setting the n-gram size between 3 and 6, and dividing the data into training, development, and test sets. Furthermore, they employ the Twitter dataset to evaluate its efficacy. The authors of [82], examine that tweets were evaluated utilizing methodologies typical of social media analytics. The pre-processing stage involves tokenization using NLTK, the emoji package, and the removal of URLs using regular expressions. In their paper, they apply the Linguistic Inquiry and Word Count (LIWC) approach and Kullback Leibner divergence for Informativeness and Phrases (KLIP) to the Twitter dataset in order to identify fake news. Another initiative [93] receives tweets that are further analyzed using single-word frequencies (unigrams) and pairs of words (bigrams). The study utilizes the LDA for point modeling to categorize the subjects discussed in the tweets. The pre-processing phase involves eliminating non-English tweets, retweets, punctuation, stop words, and non-printable characters. It also includes normalizing Twitter user mentions and lemmatizing the text.

A contemporary project [94] proposes a deep learning framework to analyze and classify Twitter users by interpreting the public graph derived from their interactions on the social network. The pre-processing stage involves content-based filtering, where two parallel layers are employed for streaming. This step entails removing poorly connected nodes and deleting them from the network, along with their linked edges. In addition, they utilize the Twitter dataset to evaluate the detection performance. By employing machine learning methods, the researchers in [95] can replicate hidden attributes of programs, such as positions and policy directives. This simulation is based on a dataset of 67 million tweets from 12 million users collected between January 29, 2020, and March 4, 2020. They categorize the users based on their countries of origin, their social backgrounds, and their political beliefs. The pre-processing stage involves testing the generalizability of the data and re-training it using geotagged information. A framework model was proposed by [98], based on the universal sentence encoder, to uncover the main trends of tweets. A universal sentence encoder is capable of extracting semantic representations and similarities between tweets. Pre-processing involves employing a sentence transformer to apply clustering methods, text summarizing, data extraction, and sentence embedding. The authors utilize the Twitter dataset from March 29, 2020, to April 30, 2020, and employ TF-IDF, LDA, and BERT algorithms to identify deceptive material pertaining to COVID-19. A comprehensive analysis of the text messages pertaining to COVID-19 has been conducted by the authors from [99]. The pre-processing stage involves AI applications, including data analytics techniques for all text messages. The utilization of AI techniques for the analysis of each tweet results in the categorization of the analyses into six distinct categories. They utilize advanced NLP templates, such as Named Entity Recognition (NER) for object recognition, Part of Speech (PoS) tagging, semantic uncertainty analysis, and text categorization. In order to validate the results, they employ widely recognized Python tools and libraries. The COV19Tweets Dataset, curated by [100], is a comprehensive collection of Twitter data. It consists of over 310 million English-language tweets specifically related to COVID-19. These tweets are accompanied by sentiment scores and originate from 204 nations and territories across the globe. The data was collected between March 20, 2020, and July 17, 2020. In a recent study, a new COVID-19 Twitter dataset consisting of 6,667 tweets was presented [96]. This dataset enables the examination and categorization of COVID-19-related discussions into five main areas: health risks, prevention, symptoms, transmission, and treatment. The results and analysis indicate that COVID-19 health-related terms in prepared datasets could have been accurately classified using a range of machine learning and deep learning algorithms. Another study [97] proposes a method that focuses on humans to identify the behavior of disseminating fake news. They develop a classifier that can detect fake news spreaders by analyzing the psychological and behavioral characteristics of individuals utilizing their own Twitter dataset. To sum up, Twitter contains a vast amount of data pertaining to the influence of COVID-19 on mental health. Researchers have collected these tweets from time to time. They have undertaken standard NLP techniques to pre-process the data. Then they employ machine learning models to track down the fake news and the impact of the fake news on public mental health. One issue to notice here is that a vast majority of the datasets were developed around 2020. Thus, we feel the necessity to build a complete and fresh Twitter dataset.

## 6 Discussion

The ecosystem responsible for propagating fake news connected to COVID-19 is significant and continues to expand. It operates in a highly collaborative manner and involves individuals who are skilled at intentionally disseminating false information. This review paper aims to comprehensively examine the field of fake news detection, including the community responsible for disseminating false information. We analyze the most widely read academic articles published between 2020 and 2024. The article focuses on the identification of false information connected to COVID-19 and the network of false news on the social media platform Twitter. We not only provide illumination on analytical publications but also provide concise summaries of dataset-sharing papers. Through the presentation of charts, methodologies, and data tables, we have determined that there exists a substantial body of research focused on identifying and combating fake news. Nevertheless, we also identified a shortcoming in the examination of the entire counterfeit news ecosystem.

*What is the novelty of this review paper?* Several systematic reviews have investigated machine learning and deep learning approaches to identify false information [101]. The review by [102] studies important publications where the authors explicitly explain fake news, outline how it spreads, and present information on the strategies and techniques employed. Another contemporary review article [103] finds a range of neural network-based classification techniques. Furthermore, the effectiveness, constraints, and difficulties of neural network methods are examined in order to classify misinformation, particularly in the context of COVID-19. Nevertheless, none of the aforementioned review papers have examined the publications related to identifying dominant fake news spreaders and their community dynamics. Conversely, none of the review articles have focused on building a large database of training datasets to detect fake news accurately and robustly. To summarize, the scope of this review paper is to examine the fake news detection models and the dataset used in those models more deeply. Therefore, we perform a more in-depth and systematic study of the detection-based work, such as identifying the dominant individuals involved in disseminating the misinformation. Moreover, we enlist a wide range of papers where the authors have created the training dataset for the detection models as well as compile these training datasets in our GitHub repository and make it public for fellow enthusiasts. Therefore, this review paper reveals significant findings, such as the continuous and fast expansion of the disseminator population, the existence of professional spreaders within this population, and a considerable level of collaboration among those who spread fake news, that will help the reader comprehensively understand the whole COVID-19-related misinformation dissemination studies.

*What are some probable future research directions?* Possible future endeavors may encompass the following:

(i) Community evolution analysis: We find no work that focuses on digging deeper into the communities being found. For example, the disseminator community evolves over time from a small to a large size. Also, the active members and their activities in communities vary from time to time. We have observed a lack of this kind of dynamic nature analysis in recent works. We can develop/improve algorithms that can handle large-scale dynamic networks efficiently. Algorithms or models like Dynamic Label Propagation Algorithm (D-LPA), Infomap, Greene's method, FacetNet, Evolutionary Spectral Clustering (ESC), Clique Percolation Method, Label Rank T, Stream-based Community Detection can be used in this regard. Also, we can develop mathematical models to describe the probabilistic nature of community changes over time. For example, we can use Dynamic Stochastic Block Model (DSBM), and Hidden Markov Model to model transitions between different community states. In addition, we can study how significant events (e.g., new variants, political elections) impact COVID-19-related community structures and dynamics using Incremental Louvain, and Bayesian Change Point Detection algorithms. Furthermore, we can investigate periods of rapid change (burstiness) and periods of stability in community structures using Incremental and Dynamic Clustering algorithms, and Cumulative Sum Control Chart methods. Moreover, the community-finding algorithms used in the existing works are very traditional. We suggest a tensor decomposition-based multi-modal analysis, which can help us gather more information about community activities over time. Additionally, profiling the communities can also be another good research direction. Some of the profiling features can be community leaders, community cohesiveness, community discussion topics, number of community members, a timespan of the communities, community engagement features, etc. These features can be presented in a human-readable visualization. There are some more research directions that are yet to be explored.

(ii) Examining the activities of purveyors of false information on alternative online platforms: The activities of the misinformation spreaders do not encompass only Twitter. The same spreader can be active across multiple social media platforms like Facebook, Reddit, etc. There is a necessity to capture their activities across platforms so that we have a full picture of their activities. One important research question can be- *How can we be sure whether two online accounts on two different platforms belong to the same person?* We can answer this research question by username matching or by profile matching. Also, we can study how misinformation spreads through networks, including the role of reposts, retweets, and shares. In the context of misinformation dissemination, we can examine cross-community interaction and influence one another as well as analyze how users migrate between platforms and how this affects the spread of misinformation. To facilitate the above-mentioned research works, we can undertake Multilayer Network Analysis, Interlayer Centrality, Cross-platform Topic Modeling via LDA, Cross-correlation Analysis, Dynamic Time Warping (DTW) Analysis, Data Fusion Techniques, Before-and-After Event Studies, etc.

(iii) Complete profiling of the spreaders: A complete and structured profiling of the disseminating individuals is needed. This will help us track the activities of the individuals across platforms, as mentioned earlier. Moreover, complete profiling can be used by law enforcement authorities to track down harmful spreaders. There are many profiling features that can be used in this direction. For example, in the context of Twitter, the number of tweets generated by the spreaders, their followers, comments, retweets, their accomplices, age, location, gender, socioeconomic status, personality traits, values and beliefs, discussion topics, text format, and stylistic features, fact-checking metrics, etc. can be good features in their profiles. Moreover, we can investigate the real-world behavioral or physical actions taken by individuals who consume misinformation, such as participating in protests, refusing vaccines, or engaging in violence.

(iv) Building a substantially large training dataset to detect fake news: Overfitting, higher prediction time, and performance concerns are some of the drawbacks we have observed with the models used for fake news identification. Scrutinizing the related works, we highly feel the necessity of building a substantially large training dataset which can be used in building powerful ML/DNN models in detecting fake news in general. In this review paper, we have built a database of training datasets so that fellow researchers can aggregate the training datasets and build a more robust and overfitting-free model to detect fake news.

*What are the limitations of our study?* While we gather, synthesize, and critically analyze a wide range of related papers, we also face some limitations. The limitations of this review paper are as follows. (a) We try to focus on COVID-19-related fake news detection models on Twitter, but in some cases, we include the works of generalized fake news detection to provide a broader view in this fake news detection domain, (b) we are able to enlist fewer efforts made in the scope of analyzing the dynamics of fake news spreaders compared to detection strategies because there are fewer efforts undertaken overall, (c) we gather and analyze only the last five years of works because our scope is COVID-19-related fake news, which was not prevalent before 2020, (d) some of the related papers may not be included in this review paper, although we tried our best to perform a wide range of search so that we did not miss any important papers on COVID-19. In defense of the above-mentioned limitation, we state that we try to focus on a particular field of interest rather than a wide one while being open to including all related papers to provide a view of the overall domain.

*Do we still need to be concerned about COVID-19-related fake news?* Although the COVID-19 pandemic era is gone, we see a surge of COVID-19 variants on a regular basis. A large portion of the world population is still not vaccinated. There are a lot of misconceptions about vaccination even in today's era. If we see the trend in Fig. 1, the number of papers published on COVID-19-related fake news each year remains flat. That means, researchers still trying to apprehend the COVID-19-related fake news propagation. There are several reasons behind their motivation. There is still the need for insights into how misinformation about COVID-19 spreads, the channels through which it propagates (e.g., social media platforms, and news outlets), and its impact on public perceptions and behaviors. There is a lack of understanding of how COVID-19-related fake news elucidates its consequences on public health outcomes, including vaccine hesitancy, non-compliance with health guidelines, and public trust in health authorities. They need to inform the development of targeted interventions and strategies to mitigate the spread of misinformation, promote accurate information dissemination, and enhance public health communication efforts. They have to develop and test innovative methodologies and advanced tools for detecting, analyzing, and monitoring fake news in real time. This includes computational techniques, machine learning algorithms, and social network analysis methods tailored to the context of COVID-19 misinformation. Scientists are innovating new drugs and vaccines regularly and new kinds of fake news are being circulated. This fake news is hindering the mass of people getting vaccines or booster doses. In summary, the researchers are publishing relevant papers currently and need to continue their work in this domain.

## 7 Conclusion

The task of addressing fake information and misleading news is becoming more crucial and challenging. The implementation of advanced machine learning and deep learning algorithms, along with the meticulous handling of datasets, have successfully tackled the issue of COVID-19 misinformation spread.

In this review paper, we gather, synthesize, and perform a comprehensive analysis of COVID-19-related fake news detection and analysis from 87 highly related research publications from a corpus of 600 over the last five years. We also discuss the methodological approaches and critically evaluate the strengths, weaknesses, and performance of the selected studies. Finally, we identify the research gaps, as mentioned in the discussion section. Moreover, going one step further, we compile 17 different training datasets in our publicly accessible GitHub repository to help the researchers utilize a large training dataset for training a robust ML model.

We also present the key findings from this review work. In the scope of fake news detection, DNN-based models, along with different embedding strategies, outperform traditional ML models. Yet, these models face challenges such as vanishing gradients, higher prediction times, and overfitting problems. Although some optimization techniques have been proposed, we feel the absence of a robust, efficient model is due to a lack of a good training dataset. Therefore, we build a database of training datasets since there is an absence of an updated one. Furthermore, in the scope of disseminator analysis, the authors have focused on identifying dominant spreaders and their communities, mostly utilizing graph modeling techniques. However, they do not investigate deeper into the communities or the dominant spreaders they find. Therefore, a wide range of potential future research directions to mitigate the research gaps have been outlined in this review paper.

In conclusion, in this review work, we comprehensively scrutinize the related works being done in the scope of identifying COVID-19-related fake news and analyzing the dynamics of the fake news spreaders. Our vital findings and recommendations have the potential to persuade the researchers and participants to address the research cavities. The preliminary results we have obtained are only the starting point of a promising future endeavor that can provide insights into this worldwide online network of false information.

## Additional information

No additional information is available for this paper.

## CRediT authorship contribution statement

**Asma Ul Hussna:** Writing – original draft, Methodology, Formal analysis, Conceptualization. **Md Golam Rabiul Alam:** Writing – review & editing, Validation, Supervision, Methodology, Formal analysis, Conceptualization. **Risul Islam:** Writing – review & editing, Software, Methodology, Formal analysis. **Bader Fahad Alkhamees:** Writing – review & editing, Formal analysis. **Mohammad Mehedi Hassan:** Writing – review & editing, Methodology, Formal analysis. **Md Zia Uddin:** Writing – review & editing, Supervision, Methodology, Formal analysis.

## Declaration of Competing Interest

The authors declare that they have no known competing financial interests or personal relationships that could have appeared to influence the work reported in this paper.

## Data Availability

Data included in article/supplementary material is referenced in the article.
